# Outer membrane vesicles secreted from *Actinobacillus pleuropneumoniae* isolate disseminating the *floR* resistance gene to Enterobacteriaceae

**DOI:** 10.3389/fmicb.2024.1467847

**Published:** 2024-09-05

**Authors:** Minsheng Xu, Haiyi Ke, Yingan Zang, Hongchao Gou, Dongxia Yang, Keda Shi, Kunli Zhang, Yan Li, Zhiyong Jiang, Pinpin Chu, Shaolun Zhai, Chunling Li

**Affiliations:** ^1^Guangdong Academy of Agricultural Sciences, Institute of Animal Health, Guangzhou, China; ^2^Guangdong Provincial Key Laboratory of Livestock Disease Prevention, Guangzhou, China; ^3^Guangdong Open Laboratory of Veterinary Public Health, Guangzhou, China; ^4^Scientific Observation and Experiment Station of Veterinary Drugs and Diagnostic Techniques of Guangdong Province, Guangzhou, China; ^5^Guangdong Gaozhou Agricultural School, Maoming, China; ^6^College of Animal Science and Technology, Zhongkai University of Agriculture and Engineering, Guangzhou, China

**Keywords:** *Actinobacillus pleuropneumoniae*, whole genome shotgun, horizontal gene transfer, resistance plasmid, outer membrane vesicles (OMVs)

## Abstract

*Actinobacillus pleuropneumoniae*, a significant respiratory pig pathogen, is causing substantial losses in the global swine industry. The resistance spectrum of *A. pleuropneumoniae* is expanding, and multidrug resistance is a severe issue. Horizontal gene transfer (HGT) plays a crucial role in the development of the bacterial genome by facilitating the dissemination of resistance determinants. However, the horizontal transfer of resistance genes via *A. pleuropneumoniae*-derived outer membrane vesicles (OMVs) has not been previously reported. In this study, we used Illumina NovaSeq and PacBio SequeI sequencing platforms to determine the whole genome sequence of *A. pleuropneumoniae* GD2107, a multidrug-resistant (MDR) isolate from China. We detected a plasmid in the isolate named pGD2107-1; the plasmid was 5,027 bp in size with 7 putative open reading frames (ORF) and included the floR resistance genes. The carriage of resistance genes in *A. pleuropneumoniae* OMVs was identified using a polymerase chain reaction (PCR) assay, and then we thoroughly evaluated the influence of OMVs on the horizontal transfer of drug-resistant plasmids. The transfer of the plasmid to recipient bacteria via OMVs was confirmed by PCR. In growth competition experiments, all recipients carrying the pGD2107-1 plasmid exhibited a fitness cost compared to the corresponding original recipients. This study revealed that OMVs could mediate interspecific horizontal transfer of the resistance plasmid pGD2107-1 into *Escherichia coli* recipient strains and significantly enhance the resistance of the transformants. In summary, *A. pleuropneumoniae*-OMVs play the pivotal role of vectors for dissemination of the floR gene spread and may contribute to more antimicrobial resistance gene transfer in other Enterobacteriaceae.

## 1 Introduction

The high infectivity of pathogens has hindered the healthy development of the global swine industry and caused incalculable economic losses (Halder et al., 2022). The effectiveness of antibiotics against drug-resistant bacteria will gradually deteriorate over time, and the abuse of antibiotics, unreasonable management methods, and the evasive mechanism of bacteria have exacerbated this phenomenon (Bonilla et al., 2022). In recent years, more and more multidrug-resistant (MDR) bacteria have been isolated and found in farms (Bentzon-Tilia et al., 2016).

*Actinobacillus pleuropneumoniae* is the etiological agent of porcine pleuropneumonia, a contagious and severe respiratory disease with high lethality, economic impact, and worldwide distribution (Bonilla et al., 2022; Zhang et al., 2018). The bacteria can cause pneumonia and sepsis in pigs, and acute symptoms can lead to the death of pigs, causing significant economic losses to the breeding industry (Da et al., 2022; Sassu et al., 2018). Since the discovery of penicillin, a large number of antibiotics have been invented and used (Da et al., 2017). However, inappropriate use has greatly accelerated the development of drug resistance of pathogenic bacteria. Florfenicol is a fluorinated structural analog of methanesulfonic acid and chloramphenicol, especially used in the veterinary field. With the increasing use of florfenicol in recent years, the resistance of *A. pleuropneumoniae* to it is increasing, and the detection rate of its key resistance gene *floR* is also increasing (Lu et al., 2018). Prevention and control of diseases in the breeding process has become more difficult.

The extensive and inappropriate use of antibiotics has fueled the spread of antibiotic resistance (AR) mechanisms in pathogenic bacteria (San et al., 2010). Bacteria acquire resistance to antibiotics through two principal routes: chromosomal mutation and the acquisition of mobile genetic elements such as plasmids by horizontal gene transfer (HGT) (Alekshun and Levy, 2007). Plasmids are circular DNA molecules that replicate independently of the chromosome and are able to transfer horizontally between bacteria by conjugation (Nang et al., 2019). Plasmids play a key role in the evolution of bacterial AR, disseminating resistance genes among the most worrisome clinical pathogens (Alekshun and Levy, 2007; Da et al., 2017). The large plasmids carrying bacterial virulence or resistance genes can be transmitted horizontally between different bacteria by conjugation (San et al., 2007).

Outer membrane vesicle (OMV) is a small spherical double-layer membrane structure with a diameter of 10–200 nm, containing bioactive substances secreted by Gram-negative bacteria during growth (Jan, 2017). In contrast to plasmids, which provide a platform for insertion sequences, transposons, integrons, and other removable elements of resistance genes, outer membrane vesicles have been studied and discovered to act as decoys to protect the parent bacteria from foreign attack, such as antibiotics, and to confer resistance to pathogens. OMV plays an important role in the survival of bacteria, including communicating with the surrounding environment, helping bacteria absorb nutrients, responding to external pressures, killing competing bacterial cells in the environment, participating in biofilm formation, and transferring biomolecules between bacterial cells (Wang et al., 2015). In addition, OMVs were also found to have the ability to horizontally transmit drug resistance genes in other strains such as *Klebsiella pneumoniae* (Lin et al., 2022), *Acinetobacter baumannii* (Chatterjee et al., 2017), *Salmonella* (Marchant et al., 2021) and *E. coli* (Yaron et al., 2000). It is suggested that OMV is involved in a number of molecular mechanisms, including the transfer of drug resistance genes at the level of drug resistance genes, the binding of pathogenic bacteria’s small molecule metabolites into the host body, and the transfer of pathogenic bacteria and host cells at the level of the bioshuttle system, where OMV functions as a functional molecule (Vanaja et al., 2016). However, whether the resistance genes could be transferred horizontally via *A. pleuropneumoniae* plasmid and OMVs remains to be unreported.

This study performed complete genome sequencing of *A. pleuropneumoniae* GD2107 (hereinafter APP-R) with florfenicol-resistant traits, and the completed sequence of the resistance plasmid was obtained. We demonstrated for the first time that OMVs are vectors of plasmids carrying florfenicol resistance genes and that they may act by transferring a functional *floR* florfenicol resistance gene between different strains of *E. coli*.

## 2 Materials and methods

The antimicrobials florfenicol were purchased from Shanghai Macklin Biochemical Co., Ltd. The culture media, pancreatic protein soybean broth medium (Tryptic soybean broth [TSB] medium) and nicotinamide adenine dinucleotide (NAD) were purchased from Beijing Suolaibao Technology Co., Ltd.; calf serum was purchased from Guangzhou Ruite Biotechnology Co., Ltd. Fisher Pierce bicinchoninic acid (BCA) Protein Assay Kit purchased from Thermo Co., Ltd. Bacterial genomic DNA extraction kit, 2 × FastTaq Premix enzyme, DL2000 DNA Marker and Protein Marker purchased from Takara Co., Ltd. All antibiotics were obtained from Hangzhou Microbial Reagent Co., Ltd. (Hangzhou, China).

### 2.1 Bacterial strains and culture conditions

Donor bacterial strains APP-R were isolated from the lungs of diseased pigs in Guangdong, China. The strain was identified by PCR amplification of its *ApxIV* genes, and further verification was performed using a homology comparison sequence. *E. coli* strain EC600 (hereinafter: EC600) and *E. coli* strain BL21-pET28a (hereinafter: BL21) were purchased from the China Center of Industrial Culture Collection. The main OMV donors for plasmid and horizontal gene transfer were strains APP-R. Strain APP-R was used as primary OMV donors for horizontal gene and plasmid transfer. Strain APP-S was used as the control strain of the OMVs donor strain. BL21 and EC600 were fully susceptible to florfenicol and used as the recipient strain. *A. pleuropneumoniae* strains were cultured in TSB medium (supplemented with 5% fetal calf serum) with 0.1% NAD, and *E. coli* strain was cultured in fresh Luria-Bertani (LB) medium and incubated at 37°C overnight.

### 2.2 Antibiotics susceptibility test

The bacterial of APP-R susceptibility to penicillin, ampicillin, amoxicillin/clavulanic acid, cefradine, cefotaxime, cefazolin, gentamicin, kanamycin, streptomycin, clarithromycin, erythrocin, azithromycin, sulfisoxazole, compound sulfamethoxazole, tetracycline, doxycycline, ciprofloxacin, enrofloxacin, florfenicol, and lincomycin acid was tested using the disc diffusion method according to clinical and laboratory standards institute (CLSI) procedures and criteria. Extended-spectrum beta-lactamases (ESBL) phenotype was confirmed using the CLSI double disc diffusion method, performed with ceftazidime, ceftazidime/clavulanate, cefotaxime, and cefotaxime/clavulanate discs, respectively. According to CLSI criteria, the results were considered positive if the diameter of the inhibitory zone increased by 5 mm in the presence of clavulanate compared with that in the absence of clavulanate.

The minimum inhibitory concentration (MIC) of florfenicol, chloramphenicol, and thiamphenicol against transformant strains was determined by broth microdilution using 96-well microtiter plates. Overnight culture of transformant strains was sub-cultured (1:100) into fresh LB media for 4 h. The bacterial density was adjusted to an OD600 of 1.0, and 100 μL bacterial suspension was incubated with various dilutions of imipenem (ranging from 0.5 to 128 μg/mL) at 37°C for 16 h. The minimum concentration of the antibiotic molecule where the bacteria fail to grow was estimated as MIC.

### 2.3 DNA extraction and whole genome sequencing

The genomic DNA of *A. pleuropneumoniae* strains was extracted using a DNA extraction kit (TaKaRa), according to the detailed protocols for Gram-negative bacteria. After detecting DNA concentration and purity, the whole genome shotgun (WGS) strategy was used to construct the libraries of PE and S10K fragments and the whole genome of the GD2107 strain was sequenced by Illumina NovaSeq and PacBio SequeI sequencing platforms, respectively. FastQc (v 0.11.7), AdapterRemoval (v 2.1.7), and SOAPec (v 2.0) software were used for quality control and filtering of sequencing data (Patel and Jain, 2012; Schubert et al., 2016). The contig sequences were assembled by HGAP (v 4) and CANU (v 1.7.1) software, and the results were corrected by pilon (v 1.22) software (Chin et al., 2016; Koren et al., 2017; Walker et al., 2014). Finally, Gview was used to construct the basic genome features (Petkau et al., 2010). Using the antibiotic resistance genes of the CARD and ResFinder databases as a query, a BLASTN search was performed against the assembled sequences of the mixed DNA with thresholds of >70% nucleotide identity and > 80% alignment coverage (Bortolaia et al., 2020; McArthur et al., 2013; Zankari et al., 2017).^1^ Mobile genetic elements were annotated using online databases, including ISfinder, INTEGRALL, and the Tn Number Registry (Carattoli et al., 2014).^2^ The complete nucleotide sequences of the chromosome and two plasmids of APP-R have been submitted to GenBank–the accession numbers of the chromosome, pGD2107-1 and pGD2107-2—are CP097377, CP097378, and CP097379, respectively.

### 2.4 OMV isolation and purification

OMVs derived from *A. pleuropneumoniae* were isolated as previously described with a slight modification (Zhu et al., 2022a). Briefly, bacteria were grown in 5-L TSB broth in a shaking incubator at 37°C for 12 h. Then bacterial suspensions were collected and centrifuged at 4°C for 20 min. Supernatants were passed through a 0.45-μm sterile filter (Millipore Corporation, USA) to remove the remaining bacteria. The dialyzed samples were concentrated to 200 mL using Amicon Ultra-15 centrifugal filter units (Merck Millipore; MWCO, 10 kDa). Following ultracentrifugation (100,000 *g*, 4 h, 4°C) of the bacterial supernatants, OMVs were resuspended in 1 × hepes buffer (pH 8.0).

To obtain more purified OMVs, gradient centrifugation using Optiprep (Sigma–Aldrich) was used (Rumbo et al., 2011). After adding crude OMVs at concentrations of about 5% 10, 20, and 40% to the top of the Optiprep gradient, the OMVs were ultracentrifuged (100,000 *g*, 10 h, 4°C) using a sw41 Ti rotor (Beckman–Coulter). To get purified OMVs, the fractions containing more vesicles were collected and subjected to additional ultracentrifugation (10 h, 4°C, 100,000 *g*). The purified OMVs were resuspended in 1.0-mL hepes buffer and stored at −80°C. The extracted OMVs were characterized by odium dodecyl-sulfate polyacrylamide gel electrophoresis (SDS-PAGE) analysis.

### 2.5 Transmission electron microscopy (TEM) and dynamic light scattering (DLS) studies

OMVs were prepared from APP-R. DLS was used to estimate the size distribution of these OMVs. The OMVs were diluted to 0.04 μg/mL in 10-mM phosphate buffer pH7.4. The scatter was recorded using a Horiba SZ-100 particle size analyzer. TEM was carried out for the OMVs using a Jeoltransmission electron microscope (JEM 2100, Tokyo, Japan) at 200 kV. The sample was loaded onto a carbon-coated copper grid, negatively stained with 0.2% uranyl acetate.

Vesicle diameter size (Z-ave) measurement and polymer dispersity index (PDI) analysis were performed using Zetasizer NanoZS (Malvern Instruments, Worcestershire, United Kingdom). 40 μL of OMVs aliquot were carefully mixed and put into sterile cuvettes for DLS. Every measurement was made at 25°C, and each purification was carried out in three separate trials. Zetasizer software (v 7.11) from Malvern Panalytical (Malvern, United Kingdom) was used to process DLS data.

### 2.6 Quantification of DNA and proteins in OMVs

OMV proteins were quantified by Thermo Fisher Pierce BCA Protein Assay Kit. For the quantification of the intravesical DNA, OMV samples were treated with DNase (Invitrogen) to hydrolyze surface-associated DNA and free DNA in suspension. Reactions were stopped by the heat treatment of the mixtures for 10 min at 65°C. DNase-treated vesicles were then lysed with 0.125% Triton X-100 solution for 30 min at 37°C, and DNA was purified by the use of a High Pure PCR product purification kit (Roche). The DNA was quantified by using a BioTek Epoch-2 instrument. The ratio of DNA to μg of protein vesicle was determined. The resistance genes carried by APP-R OMVs were identified using PCR assay. Approximately 3 μL of Proteinase K (PK)/deoxyribonuclease I (DNase I)-treated OMVs produced by strains APP-R were used as PCR templates.

### 2.7 Plasmid extraction and transformation

Based on the results of WGS, integron-negative APP-R were used as donor strains that provided drug-resistance genes to the recipient strains. BL21 and EC600 strains, sensitive to florfenicol, were used as recipient strains to accept and integrate resistance genes. The plasmid of APP-R strains was extracted using a plasmid extraction kit (Omega) according to the detailed protocols for Gram-negative bacteria. A plasmid conjugation transfer assay followed a published method (Lomovskaya et al., 2017). The plasmid extracted from 1 μL was added to the prepared, competent cells, then clicked with 1.8 KV voltage, and then added to the TSB medium preheated by 1 mL. Then transferred to a 1.5-ml centrifuge tube and placed in a shaker in 160 ~ 225-rpm resuscitation for 1.5 h. The transformation product of 100 μL was spread evenly on the surface of the whole resistant medium (florfenicol [FFC]/Tryptic soy agar [TSA]) with a sterile glass coating stick. Under 37°C, the colony growing in the resistant medium was cultured overnight. Once the experiment was performed in triplicate, the conjugation frequency (*F*_c_) was calculated according to *F*_c_ = *T/R*, in which *T* represents the number of transconjugant colonies and *R* represents the number of recipient isolate colonies. Finally, the transconjugant colonies were tested by PCR to determine whether the resistance genes were transferred to the recipient isolates.

### 2.8 OMVs mediate the plasmid interspecific transfer

With slight modifications, the OMVs-mediated resistance plasmids transfer was measured as previously described (Bielaszewska et al., 2020). For resistance plasmid transfer through OMVs, the recipient *E. coli* strains (BL21 and EC600) were grown in LB medium, reaching OD_600_ of 0.4, and the concentration was adjusted to 1.0 × 10^8^ CFU/mL. Recipient bacteria suspensions (100 μL, 1.0 × 10^7^ CFU) were incubated statically with 50 μg of PK/DNase I-treated APP-R OMVs at 37°C for 4 h and then incubated at 37°C on a shaker at 180 rpm for 4 h. After the above steps, the incubated samples were added with LB broth to 10 mL and incubated overnight (37°C, 180 rpm). The next day, bacteria suspensions were plated on LB agar plates with 8 μg/mL concentrations of florfenicol. To find out whether APP-R OMVs could transfer the floR gene and to determine the OMV-DNA amount required, EC600 and BL21 were incubated with increasing doses of PK/DNase-treated APP-R OMVs-LB and the frequencies of floR transfer were calculated by dividing the number of vesicants (CFU/mL on ESBL agar) by the total bacterial count (CFU/mL on LB agar).

### 2.9 Characterization of the transformants

The transformants were subjected to a series of analytical procedures to ascertain their characteristics. The primers for PCR reaction in 20 μL volumes are listed in Table 1. The presence of *floR* genes was confirmed in the transformants by PCR. Other primer information is shown in Table 1. The minimum inhibitory concentration (MIC) values (in μg/mL) of various antibiotics were determined by Etests in the transformants.

**Table 1:** Oligonucleotide sequences used as PCR primers.

Gene	Primers	Sequence (5′-3′)	Reference
*floR*	*floR* forward	CGACTCCATCCATAATCCAA	This study
	*floR* reverse	AACGAAGAAGGTGCCTATAC	
*ApxVI*	*ApxVI* forward	TTGGTTTAGCCTTATCCGAACT	Zhang et al. (2019)
	*ApxVI* reverse	AACCATCCGTCCATATTTGATAA	
*16S rRNA*	*16S rRNA* forward	CATGAAGATGCGGACTTGCG	This study
	*16S rRNA* reverse	GCTAACGTATCCACGCCGTA	

The gene“floR,”“ApxVI” and “16S rRNA” are, respectively, detection for florfenicol resistance genes, *A. pleuropneumoniae*, and *E. coli* strain.

### 2.10 Growth curve and growth competition experiments

Growth kinetics were determined for BL21 and BL21-Tc, and EC600 and EC600-Tm. Volumes of 30-ml LB broth were inoculated independently with 10^7^ CFU of BL21 and BL21-Tc, EC600, and EC600-Tm; cultures were grown for 12 h at 200 rpm and 37°C. The absorbance at 600 nm was measured every hour. The fitness cost of pGD2107-1 was determined between BL21 and BL21-Tc, EC600 and EC600-Tm, as previously described (Li et al., 2022), with the following modifications. BL21 and BL21-Tc, EC600 and EC600-Tm, were cultured in LB broth for 24 h at 37°C and 200 rpm. Then 1 × 10^8^ CFU of recipient strain was mixed with 1 × 10^8^ CFU of the corresponding transconjugant/transformant in 30-ml antibiotic-free LB broth. The mixtures were grown at 37°C and 200 rpm and diluted at 1:100 to fresh LB broth every 12 h. For each sample, aliquots were plated onto non-selective and florfenicol-containing LB agar plates. The percentage of strains harboring the pGD2107-1 marker was calculated by dividing the number of colonies developing on the selected (positive) plates by the total number of colonies present on both positive and negative plates.

## 3 Results

### 3.1 Antibiotic resistance

The drug-sensitive test was used to determine the resistance of the drug to bacteria. APP-R was resistant to florfenicol, chloramphenicol, and so on. BL21 and EC600 was susceptible to florfenicol (Table 2). The results indicated that APP-R might carry the florfenicol-resistant genes.

**Table 2:** MIC values of antibiotics tested in this study.

Strains		Drug resistance determination										
		FFC	CHL	THI	TET	GEN	AMP	PIP	CIP	NOR	SUL	AMO
Donor strain	APP-R	R	R	R	R	I	S	R	R	R	R	S
Recipient strain	BL21	S	S	S	S	S	S	S	S	S	S	S
	EC600	S	S	S	S	S	S	S	S	S	S	S

AMO, amoxicillin; AMP, ampicillin; CHL, chloramphenicol; CIP, ciprofloxacin; FFC, florfenicol; GEN, gentamicin; I, intermediary; ND, not determined; NOR, norfloxacin; PIP, piperacillin; R, resistant; S, sensitive; STR, streptomycin; SUL, sulfisoxazole; THI, thiamphenicol; TET, tetracycline.

### 3.2 Whole genome sequencing of GD2107

The whole chromosomal and plasmid sequences of APP-R were sequenced using Illumina NovaSeq and PacBio sequencing technologies. As shown in Supplementary Figure S1, this strain had two plasmids named pGD2107-1 (5,027 bp, Genbank:CP097378) and pGD2107-2 (3,497 bp, Genbank:CP097379), as well as a chromosomal genome (2,271,987 bp, Genbank:CP097377), according to the sequence analysis of the GD2107 complete genome. The whole genome, according to sequencing data, reveals the florfenicol resistance gene floR is carried by the plasmid pGD2107-1. Any other resistance genes, such as sul, tet, and so on, are located on bacterial chromosomes (Supplementary Figure S1A; Supplementary Table S1). The plasmid pGD2107-1 contained a conjugal transfer region and the floR gene, conferring florfenicol resistance (Supplementary Figure S1B). The multidrug resistance of APP-R was further amplified by the presence of this resistance plasmid. It is notable that no other resistance or virulence genes are present in this plasmid (Supplementary Figure S1C). Therefore, pGD2107-1 stands as a valuable resource for the verification of florfenicol resistance transfer.

### 3.3 Plasmid structures

**Figure 1:**
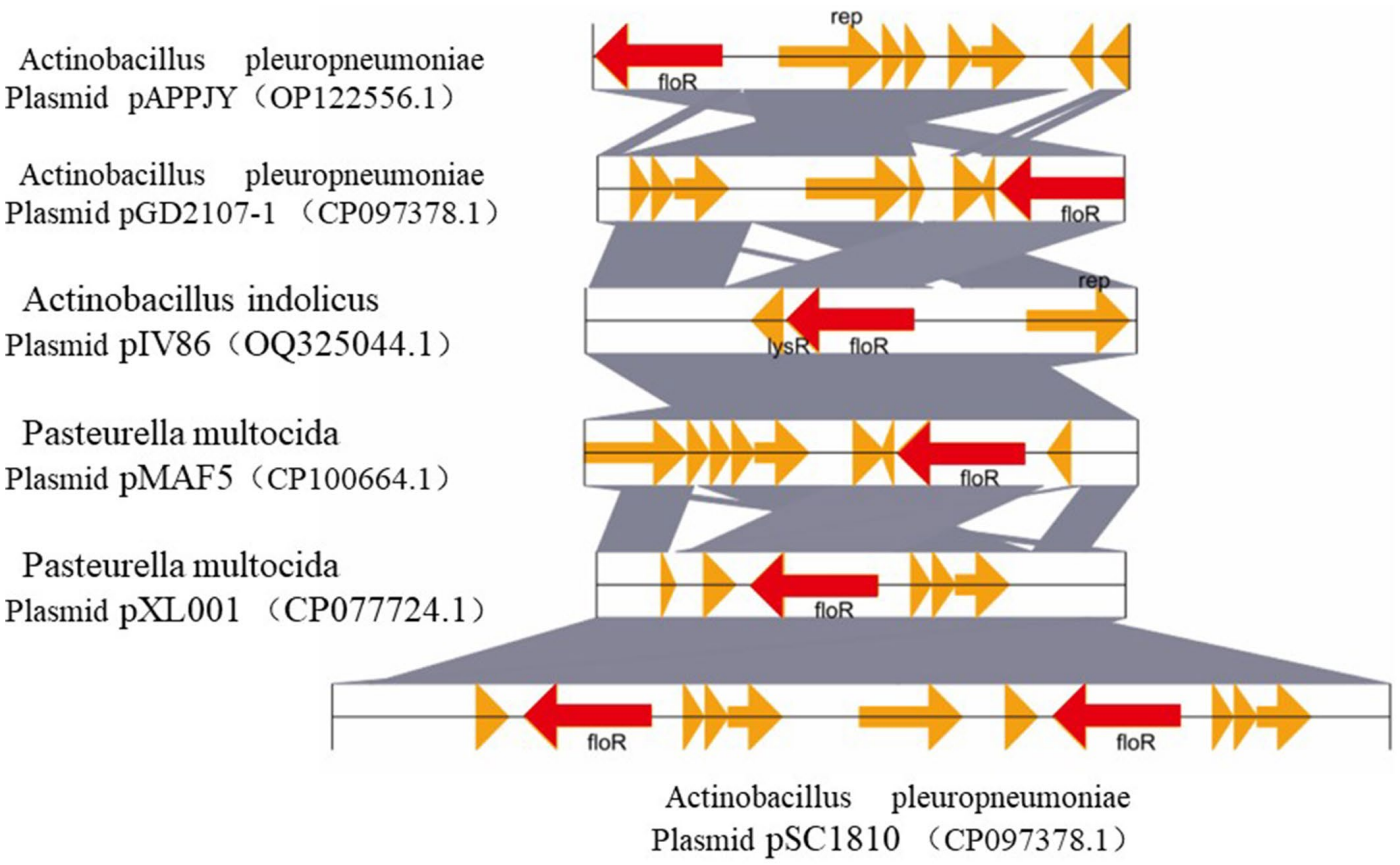
Comparative structural analyses of pGD2107-1 and other plasmids. Genetic environment and comparative analysis of the *floR* gene-related region in plasmid pGD2107-1 of *Actinobacillus pleuropneumoniae*. OQ325044.1, the plasmid pIV86 of *Actinobacillus indolicus* strain IV86 isolated from a lung of a swine in Fujian, China; CP100664.1, the plasmid pMAF5 of *Pasteurella multocida* strain XN-PM-1 isolated from a swine in the Shanghai, China; CP077724.1, the plasmid pXL001 of *P. multocida* strain PMWSG-4 isolated from a liver of a duck in Guangdong, China. OP122556.1, the plasmid pAPPJY of *A. pleuropneumoniae* strain AppJY isolated from a lung of a sick pig in Jiangsu Province, China; CP071699.1, the plasmid pSC1810 of *A. pleuropneumoniae* strain SC1810 isolated from a swine in Sichuan Province, China. The homologous genes present in the five sequences are marked with the same colors, and the non-homologous genes are in gray.

The drug-resistant plasmids obtained by sequencing were compared with the Basic Local Alignment Search Tool (BLAST) on the National Center for Biotechnology Information (NCBI), the other five similar plasmids were analyzed and compared, and the structural comparison diagram was drawn (Supplementary Table S2). It can be seen from Figure 1 that these six plasmids have similar skeleton structures, and more than 90% homology indicates that there may be a certain relationship between them. Whole genome sequencing, assembly, and analysis for *A. pleuropneumoniae* GD2107 showed a 5,027-bp small plasmid (designated pGD2107-1) harbored the floR gene, with an average GC content of 42.28%. A total of seven open reading frames (ORFs) were identified, each encoding a protein of >100 amino acids. The plasmid mobilization protein encoded by the floR gene from pGD2107-1 exhibited a high level of homology (identity ≥94.95%) to that encoded by floR genes from various *Pasteurella multocida* strains, including CP100664.1, CP077724.1, OP122556.1, and CP071699.1. Additionally, it demonstrated significant similarity to the protein from *Actinobacillus indolicus* (OQ325044.1). The replication protein encoded by the rep gene from pGD2107-1 exhibited a sequence identity of ≥99.5% to that encoded by rep genes from *A. pleuropneumoniae* AppJY (OP122556.1). Furthermore, the floR-carrying pGD2107-1-like plasmid was found to be widespread, as evidenced by its presence in *A. indolicus* strain IV86 (OQ325044.1) (Figure 1).

### 3.4 Characterization of isolated *A. pleuropneumoniae*-OMVs

**Figure 2:**
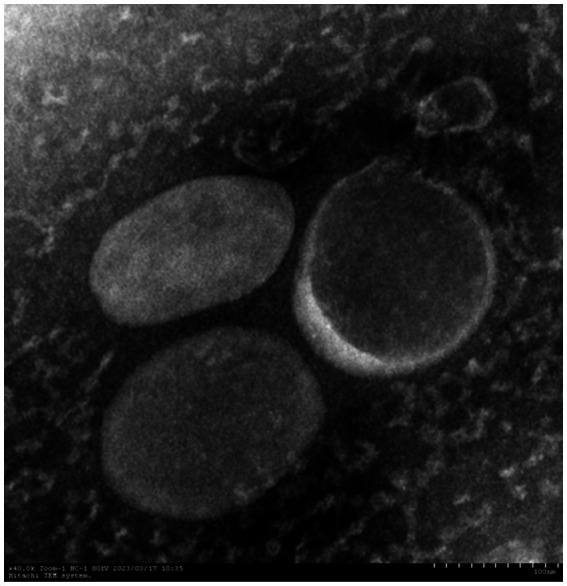
Electron microscopy of OMVs.

We evaluated the OMVs under research in terms of morphology, size, and polydispersity index (PDI) using culture supernatants in order to highlight some of their characteristics. At TEM, pure OMVs were observed as uniformly spherical, electron-dense particles (Figure 2). We verified the total sterility of the vesicular suspensions by adding purified vesicles to the TSB medium (supplemented with 5% fetal calf serum) with 0.1% NAD. The results showed no bacterial growth in the suspension.

**Figure 3:**
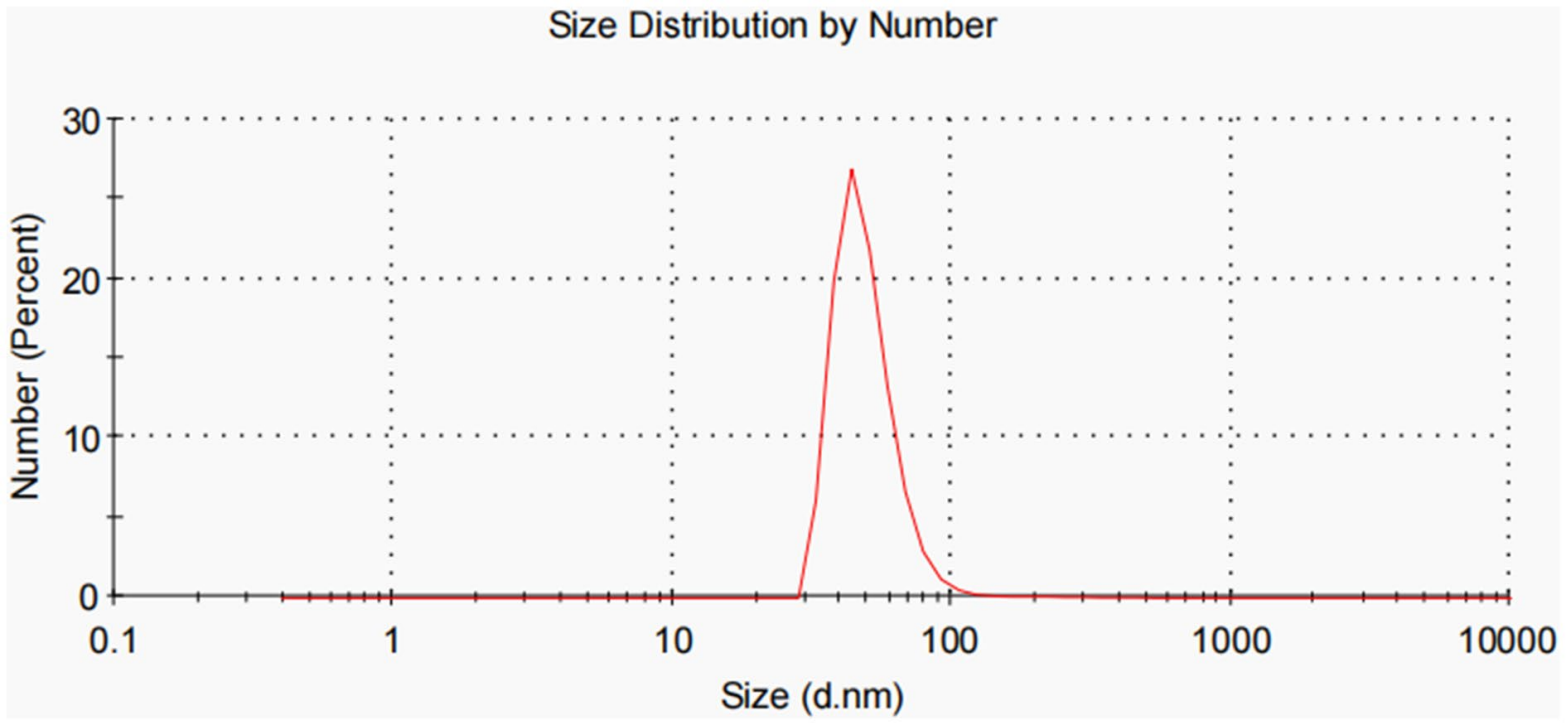
Particle size distribution (PDI) of OMVs.

All experiments were performed in triplicate and repeated thrice. Dynamic light scattering (DLS) analysis showed that OMVs derived from APP-R measured a size of 50.53 ± 24.09 nm and were characterized by a slightly heterogeneous size distribution, represented by the PDI of 0.461 (Figure 3). The OMVs of *A. pleuropneumoniae* strains were heterogeneous and exhibited multiple spherical vesicles with a diameter range of 20–100 nm.

### 3.5 Isolation and purification of OMVs from APP-R

**Figure 4:**
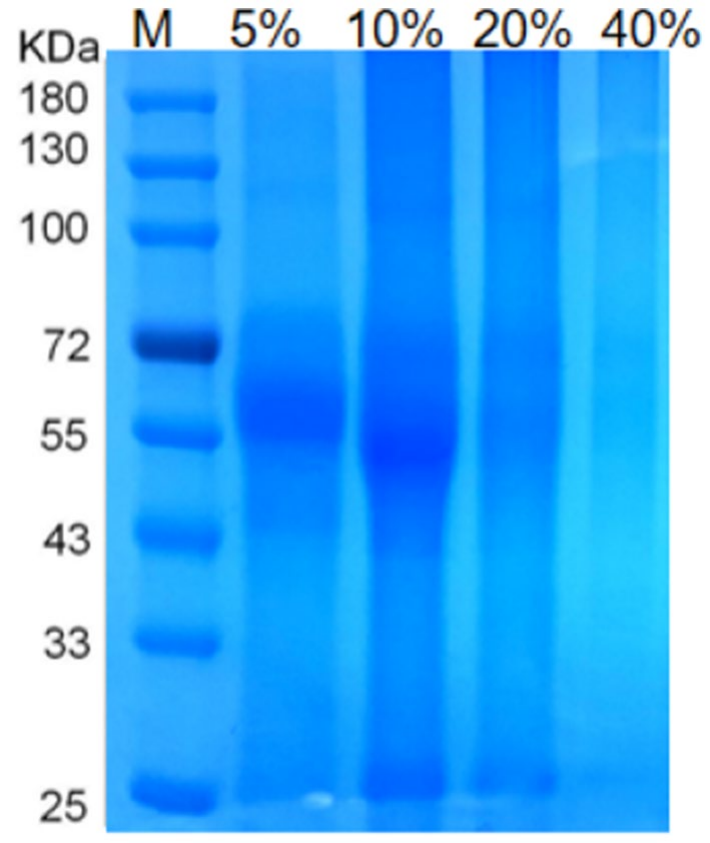
The results of OMVs density gradient centrifugation SDS-PAGE. *Note:* Protein profile of OMVs of *A. pleuropneumoniae* SDS-PAGE of OMVs extracted from different separation liquid concentrations. Lane M contains molecular mass standards.

OMVs production of *A. pleuropneumoniae* at 12 h (early stationary phase) culture time and it was acquired from cell-free supernatants by ultracentrifugation. Then, to obtain the purer OMVs, vesicles were purified, and impurities in *A. pleuropneumoniae* OMVs were eliminated using density gradient ultracentrifugation (DGU). Moreover, the concentration of the purified OMVs of *A. pleuropneumoniae* was quantified using a protein detection kit. The concentration of OMVs, collected from 2 L culture and resuspended in 1.0 mL, was approximately 9.1 × 10^11^ particles/ml. The protein concentration of OMVs for various concentrations of Optiprep was measured as 1.5 μg/μL, 2.2 μg/μL, 1.2 μg/μL, and 0.4 μg/μL. This meant that the highest content was 2.2-μg OMVs collected from 10% of separation liquid concentration after *A. pleuropneumoniae* growth for 12 h. The total DNA of PK/DNase-treated OMVs was measured, and the DNA content in 9.1 × 1,011 OMVs cargo was approximately 5.06 ng of DNA. These results meant that the DNA concentration in OMVs was about 5.06 (Table 3). The SDS-PAGE experiments showed the concentration of OMV at different concentrations of separation solution (Figure 4). Through SDS-PAGE experiments and protein concentration test results, we chose to use OMVs with a separation liquid concentration of 10%.

**Table 3:** The protein concentration of OMV in different separation solutions.

Separation liquid concentration (5)	μg of vesicle protein/μL	μg of DNA/μg of vesicle protein
5	1.5	5.06
10	2.2	
20	1.2	
40	0.4	

### 3.6 Detection of resistance genes in the OMVs and plasmid of APP-R

**Figure 5:**
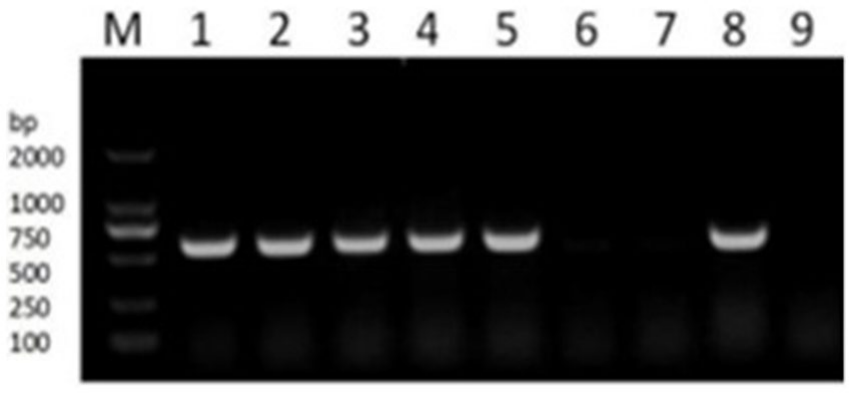
PCR verification of drug resistance gene *floR.* PCR detecting the resistance genes *floR* (lane 1–6). M, DL2000 marker; lane 1, the genome of APP-R; lane 2–3, plasmid; lane 4–5, OMV; lane 6–7, BL21 and EC600; lane 8–9, positive and negative.

The presence of resistance genes in donors and recipient isolates was detected by PCR testing. Florfenicol-resistant APP-R carried resistance gene floR, BL21, and *E. coli* 600 were sensitive to florfenicol and had no resistance gene floR. To analyze whether OMVs carrying resistance genes and OMVs derived from strains APP-R were used for PCR testing. Our result showed resistance genes could be detected in APP-R, plasmid, and OMVs (Figure 5). Our results also indicated that DNA was packaged in OMVs produced by *A. pleuropneumoniae* strains APP-R. Other strains did not carry the resistance gene floR.

### 3.7 APP-R OMVs simultaneously transfer resistance plasmids into BL21 and EC600 recipient strains

**Figure 6:**
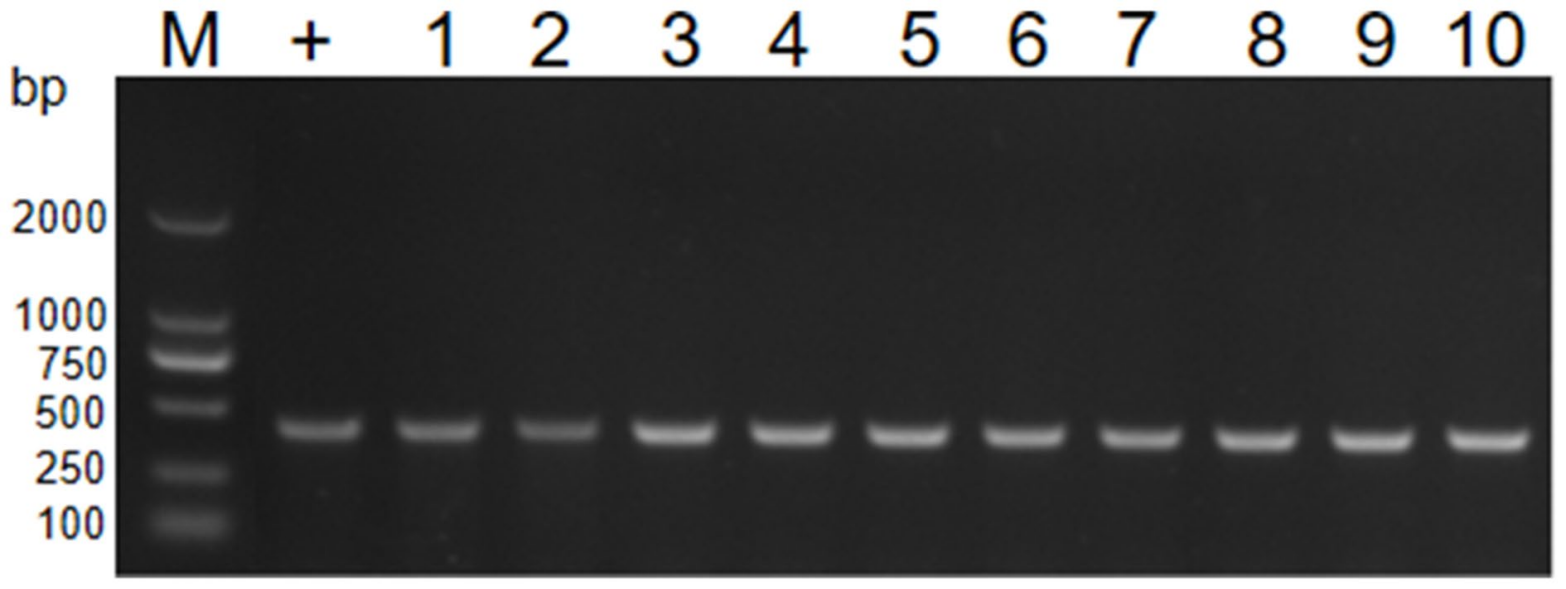
PCR verification of transformant by *16S rRNA*. PCR detecting the resistance genes16S RNA of *E. coli* (lane 1–10). M, DL2000 marker; lane 1–5, the genome of BL21; lane 6–10, the genome of EC600.

For the transfer of resistance plasmids, GD2107 acted as the donor strain, and strains BL21 and EC600 were used as recipient strains. Colony–PCR was performed to detect the resistant acquisition (Figure 6). No plasmid acquisition happened when recipient strains were treated with free plasmid OMVs (Date not shown). For recipient strain BL21 and EC600, after incubation with 50 μg of APP-R OMVs, the transformation frequencies were 8.2 ± 0.3 × 10^4^ CFU/μg DNA and 4.5 ± 0.6 × 10^4^ CFU/μg DNA, respectively. The transformation frequencies of the two recipient isolates, *E. coli* BL21 and *E. coli* EC600 were 2.94 × 10^−4^ and 3.02 × 10^−4^, respectively (Table 3). With regard to the experimental conditions, the transformation efficiency of OMV is more efficient than plasmid, as it does not necessitate external experimental conditions such as thermal stress and electric shock. Similar trends were shown in the study conducted by Dell Annunziata et al. on OMVs derived from *K. pneumoniae* (7.9 ± 0.3 × 10^4^ CFU/μg DNA) (Dell'Annunziata et al., 2021). Our results had a higher transformation efficiency than other studies in which OMVs were derived from *E. coli*, *Salmonella enterica*, *Pseudomonas aeruginosa*, and *B. cepacia* with a transformation efficiency of 1.7 ± 0.2 × 10^4^, 1.5 ± 0.9 × 10^4^, 1.6 ± 0.1 × 10^4^, and 1.8 ± 0.8 × 10^4^ CFU/μg DNA, respectively (Table 4).

**Table 4:** The results of conjugation frequencies of BL21 and EC600.

Donor	Recipient	Resistance gene transferred	Conjugation frequency
GD2107-plasmid	BL21	*floR*	2.94 × 10^−4^
	EC600	*floR*	3.02 × 10^−4^
GD2107-OMV	BL21	*floR*	8.2 ± 0.3 × 10^4^
	EC600	*floR*	4.5 ± 0.6 × 10^4^

### 3.8 Characterization of the transformants

**Figure 7:**
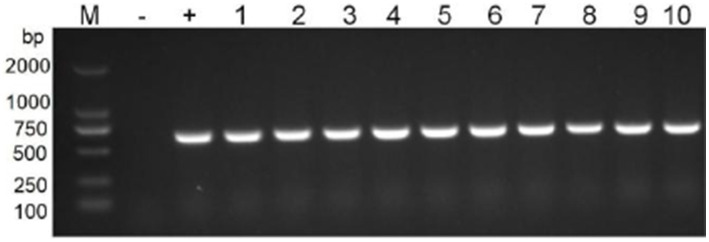
PCR detection of resistance genes floR in transformants. M, DL2000 marker; lane 1–5, nuclein and bacteria of BL21; lane 6–10, nuclein and bacteria of EC600.

Transformants were identified by PCR, and their resistance contrasts were determined by MIC values and phenotypic tests. Transformants BL21 and *E. coli* selected for characterization were positive for the *floR* genes (Figure 7). The clinical strain showed a broad profile of chloramphenicol resistance, including florfenicol, chloramphenicol, and thiamphenicol. Both recombinants (BL21 + pGD2107-1 and EC600 + pGD2107-1) showed increased MIC levels (at least 32-fold) for florfenicol, thiamphenicol, and chloramphenicol compared with the recipient strain (Table 5). The alteration in MIC values indicated the successful transfer of a functional *floR* gene, accompanied by the active expression of the chloramphenicolase in the respective recipients.

**Table 5:** The results of drug resistance phenotype of bacteria BL21 and transformants.

Antibiotic	MIC(μg/ml)			
	BL21	BL21 + pGD2107-1	EC600	EC600 + pGD2107-1
Florfenicol	<1	64	<1	128
Chloramphenicol	2	32	2	64
Thiamphenicol	4	>256	2	>256

### 3.9 Fitness cost

**Figure 8:**
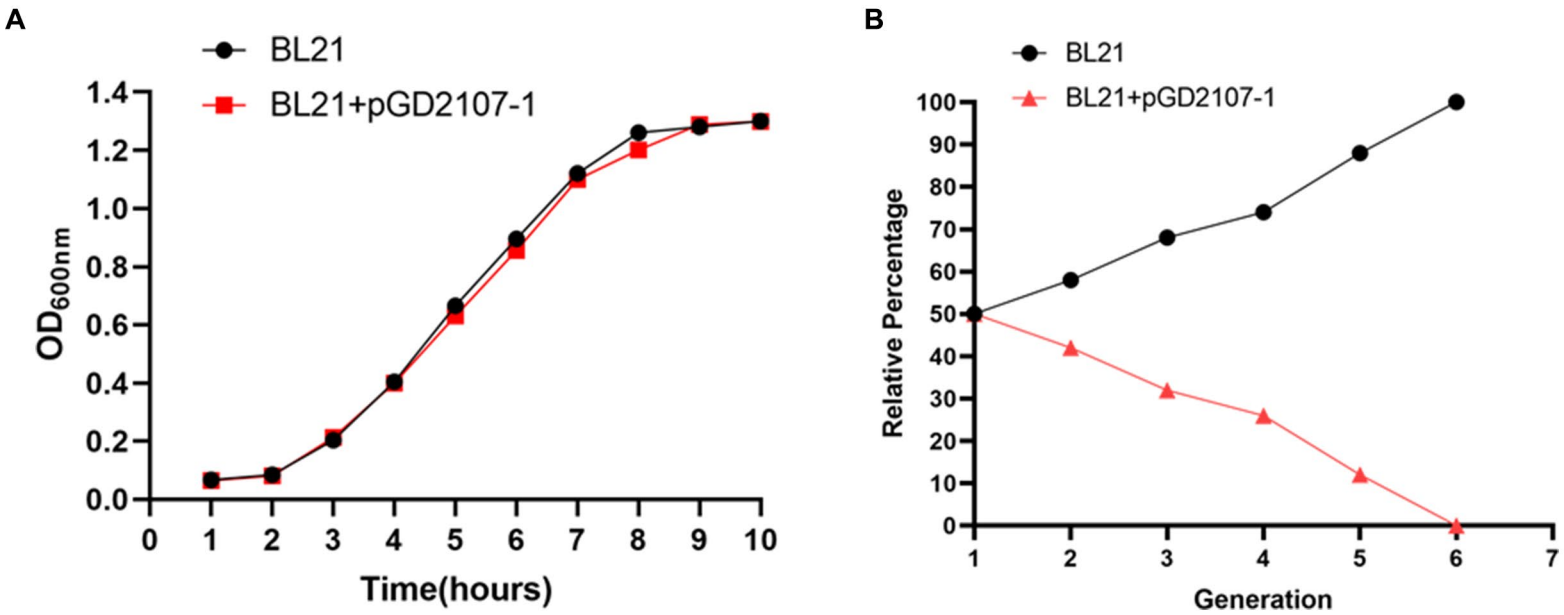
Fitness cost of BL21 and transformants. **(A)** Growth curve of *E. coli* BL21 and transformant BL21 + pGD2107-1; **(B)** competition test between *E. coli* BL21 and transformant BL21 + pGD2107-1.

**Figure 9:**
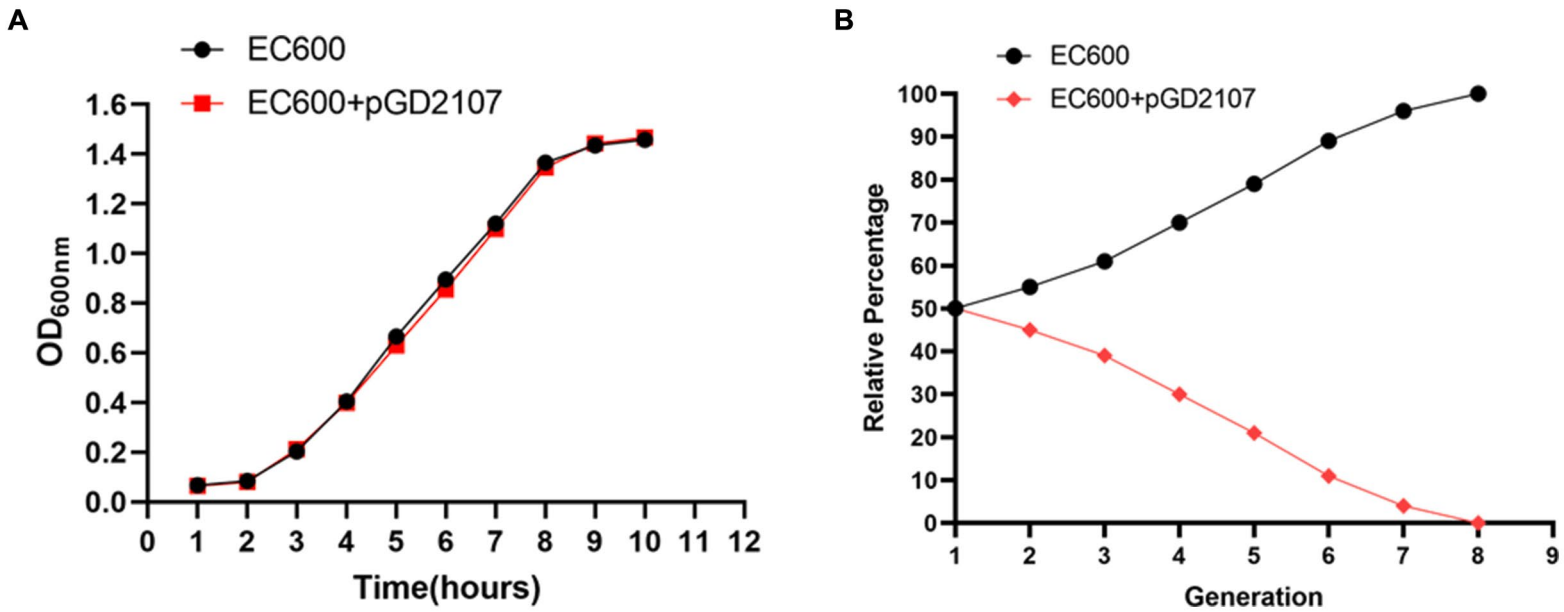
Fitness cost of EC600 and transformants. **(A)** Growth curve of *E. coli* EC600 and transformant EC600 + pGD2107-1; **(B)** competition test between *E. coli* EC600 and transformant EC600 + pGD2107-1.

The growth curve of BL21, EC600, and their transconjugants and transformants in the absence of florfenicol are shown in Figures 8, 9. The findings showed that in the absence of selection pressure, there was no discernible difference in the fitness of transformants and transconjugants bearing pGD2107-1 as compared to recipient strains. The recipient strain that had pGD2107-1 had a 1:1 starting ratio with the original recipient strain. The aforementioned results were averaged from four independent experiments.

Fitness may be measured more precisely and selectively through competition trials, and the fitness load imposed by pGD2107-1 may create a competitive disadvantage throughout the growth cycle and in cycles that follow. The fraction of *E. coli* BL21-Tc was shown to gradually decline during the competition trial between BL21 and BL21-Tc starting on day 1, and all of the examined colonies were pGD2107-1 free on generation 7 (Figures 8A,B). From the outset, a rapid and consistent decline in the prevalence of *E. coli* EC600-Tm was evident, with all strains subjected to pGD2107-free testing in generation 8 (Figures 9A,B). The aforementioned results indicated that all the pGD2107-1-carrying transconjugants and transformants exhibited a fitness cost in comparison to the recipient strains devoid of pGD2107. However, the extent of the fitness cost varied among the diverse transconjugants and transformants.

## 4 Discussion

In recent years, the extensive use of antibiotics worldwide has led to an increase in the number of drug-resistant bacterial strains, thus resulting in an increasingly severe degree of bacterial resistance (Liu B. G. et al., 2022). When bacteria frequently acquire resistance genes, it will lead to treatment failure further affecting farming production. *A. pleuropneumoniae* is an important porcine bacterial pathogen widely distributed in nature. It is one of the most common pathogens of disease outbreaks in the breeding industry (Liu F. et al., 2022; Luan et al., 2022). Compared with other pathogens causing pig diseases, *A. pleuropneumoniae* is mainly targeted at fattening pigs. This study focused on the dissemination of resistance genes among *A. pleuropneumoniae* strains and other Enterobacteriaceae strains, such as *E. coli* strain EC600.

The exchange of genes, particularly those conferring antibiotic resistance, is a common phenomenon observed among distantly related species, leading to the rapid spread of resistance (Hegstad et al., 2020). HGT plays an essential role in bacterial genome evolution by spreading antibiotic resistance and virulence determinants (Lu et al., 2017). It is well-known that transformation, transduction, and conjugation are the three classic mechanisms that promote bacterial horizontal gene transfer among intraspecies or interspecies. Thus, it can promote bacterial evolution, adaptation to environmental changes, and acquisition of new metabolic capabilities (Lerminiaux and Cameron, 2019; Lu et al., 2017). OMVs are the latest proposed type in HGT mechanisms. Several studies have reported that OMVs carrying resistance and virulence genes have the potential to deliver them to other bacteria but not include *A. pleuropneumoniae* (Bielaszewska et al., 2020; Kolling and Matthews, 1999; Wang et al., 2022). The role of plasmid and OMVs isolated from *A. pleuropneumoniae* in the transfer of *floR* is the focal point of this study. Through this study, we hope to verify whether the *floR* gene can be transformed and transmitted to other Enterobacteriaceae strains through plasmids and OMVs. An improved understanding of *A. pleuropneumoniae* mechanisms to resistance genes spread is needed to limit such a serious threat to the global breeding industry. To our knowledge, this is the first report that *A. pleuropneumoniae*-derived OMVs could carry and transport resistance plasmid, involved in the sensitivity of *A. pleuropneumoniae*, into multidrug-resistant strains.

First, we performed PCR identification and drug sensitivity tests on the clinically isolated strains and found that they showed multidrug resistance phenotype (Table 2). The complete plasmid sequences of APP-R were revealed by Pacbio sequencing. Strain GD2107 contains two plasmids, one of which plasmid (pGD2107-1) carried the antibiotic resistance gene for florfenicol. It has been postulated that large plasmids are readily transferable between closely related species (Dell'Annunziata et al., 2021). Li et al. reported that a plasmid pHPSF1 carrying floR gene expression and resistance to florfenicol was identified in *Haemophilus parasuis*. Through structural alignment, it was found that it was highly similar to other plasmids derived from *Pasteurella*, indicating that the plasmids of *Pasteurellaceae* species were more inclined to exchange genetic elements (Li et al., 2015). In this study, pGD2107-1 was highly similar to the plasmid pAPPJY and pSC1810 isolated from *A. pleuropneumoniae.* And the plasmids pMAF5, pXL001, and pIV86 were isolated from *A. indolicus* and *Pasteurella muitocida.* The skeleton structure has high similarity, but there are differences in some functional genetic elements. It is speculated that the plasmid is accompanied by the evolution of genetic elements during the transfer process. The plasmid of the resistance gene *floR* isolated from *A. pleuropneumoniae* was transferred into *E. coli* for the first time and the MIC of the transformant was increased, which verified that the plasmid pGD2107-1 had the function of mediating the resistance of bacteria to chloramphenicol antibiotics. At the same time, there is a potential evolutionary relationship between plasmids, suggesting that further monitoring studies of florfenicol resistance and the prevalence of the *floR* gene are necessary not only for *A. pleuropneumoniae*, but also for other species associated with porcine respiratory infections such as *A. indolicus* and *P. muitocida.*

Subsequently, the transfer frequency of OMV-mediated pGD2107-1 into different recipient cells was identified. OMV was isolated from *A. pleuropneumoniae* using TEM, and DLS analysis showed that the vesicles were spherical with a diameter of 20–200 nm. Fulsundar et al. showed that the different vesicular size, production, and DNA content determined a significant effect such as antibiotic and environmental stresses during bacterial growth (Fulsundar et al., 2014; Juodeikis and Carding, 2022). These data demonstrate that the release of OMVs is a physiologically regulated process that is influenced by external circumstances. OMVs were double-layer membrane vesicles packaged by lipopolysaccharides and phospholipids, which were similar to the host bacteria (Shi et al., 2022). PCR detection also found that the APP-R strain and the extracted plasmid carried the florfenicol resistance gene *floR*. It has been reported that large plasmids are believed to be easily transferable between closely related species (Dell'Annunziata et al., 2021). Evidence suggests that there exists DNA (plasmid or genome fragments) on the surface of OMVs (Rodriguez and Kuehn, 2020). Our study identified that virulence and resistance genes could be packaged into OMVs derived from APP-R. It is well established that the ability of plasmids to transfer to recipient cells is contingent upon the specific types of plasmid replicons involved, with incompatibility between replicons being a key factor (Wang et al., 2022). This is, to the best of our knowledge, the first report that OMVs, emerging vehicles of ARG transfer, carry and disseminate the *floR* resistance gene among clinically important Enterobacteriaceae. This substantially extends the currently known mechanisms of the horizontal *floR* transfer. The observation is in agreement with OMV-mediated transfer of ARG-containing plasmids in other bacterial species (Bielaszewska et al., 2020; Rumbo et al., 2011; Yaron et al., 2000). We determined that OMVs held the high efficiency of resistance plasmid horizontal transfer into recipient strains. This study provided a view that plasmid-carrying resistance genes could be transferred horizontally via OMVs into Enterobacteriaceae. Nowadays, there are more and more reports about the emerging and spread of florfenicol-resistant hypervirulent *A. pleuropneumoniae* (Juodeikis and Carding, 2022; Negrete-Abascal et al., 2000; Zhu et al., 2022). OMVs-mediated plasmid transfer might promote the emergence of florfenicol-resistant *A. pleuropneumoniae* and be involved in clinical infections in animals. However, there were two limitations in this study, including the small *A. pleuropneumoniae* samples and host sources, and we just assessed the OMVs-mediated plasmid transfer in two types of Enterobacteriaceae strains. In future research, we would evaluate the OMVs-mediated plasmid horizontal transfer of *A. pleuropneumoniae* strains from other sources, such as poultry and companion animals.

In this context, our study showed that *A. pleuropneumoniae* transferred genetic material by integrating DNA within the OMVs, and by determining the acquisition of resistance genes present in the plasmid, the DNA in the vesicular lumen was transferred to the target cell. The recipient bacterium *E. coli* 600, after contact with OMVs, acquired and expressed resistance to florfenicol, proving the OMVs’ ability to promote intraspecies HGT. When cells were incubated without plasmid, plasmid transfer did not occur, indicating that vesicles may represent a physiological mechanism that surpasses environmental limitations (dilution of gene material, exonuclease degradation, long-distance transfer, etc.) and is linked to the donor/recipient cell (high vesicle-OMV affinity, correlation phylogenetics, etc.). Chatterjee et al. have already reported the ability of *A. pleuropneumoniae*-OMVs to allow interspecies gene transfer (Chatterjee et al., 2017). The experimental evidence demonstrated that OMVs facilitated genetic exchange in microbial communities, even among distantly related bacteria, without the involvement of specific exchange mechanisms. Future studies will examine the potential of OMVs for DNA exchange between different Gram-positive species.

## 5 Conclusion

In summary, the present study demonstrates once again OMVs-mediated plasmid spread resistance gene *floR* was an important plasmid transfer mechanism to promote the dissemination of resistance genes among Enterobacteriaceae. This specific HGT mechanism permits diffusion within or between species, persisting over time and not requiring any particular conditions. Furthermore, the transmission of resistance genes amongst various bacterial species could be facilitated by this process. It may be possible to find novel pharmaceutical targets to stop the emergence of antibiotic resistance by better understanding the distribution of resistance determinants in various bacterial populations and elucidating the conditions that encourage their expansion.

## Data Availability

The datasets presented in this study can be found in online repositories. The names of the repository/repositories and accession number(s) can be found in the article/Supplementary material.

## Footnotes

^1^ https://cge.cbs.dtu.dk/services/ResFinder/

^2^ https://isfinder.biotoul.fr/
