# Supplementary material for: Outer membrane vesicles secreted from Actinobacillus pleuropneumoniae isolate disseminating the floR resistance gene to Enterobacteriaceae
Source: Front Microbiol. 2024 Sep 5;15:1467847. doi: 10.3389/fmicb.2024.1467847 (PMC11410613; doi:10.3389/fmicb.2024.1467847)
Supplement: Supplementary file 1 [file Data_Sheet_1.docx]

# Supplementary Material

**Figure S1: Gene completion maps of bacteria and plasmids**


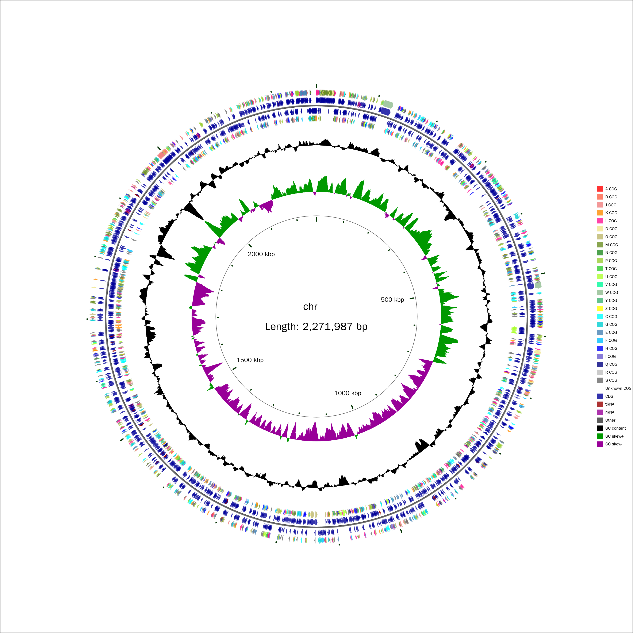

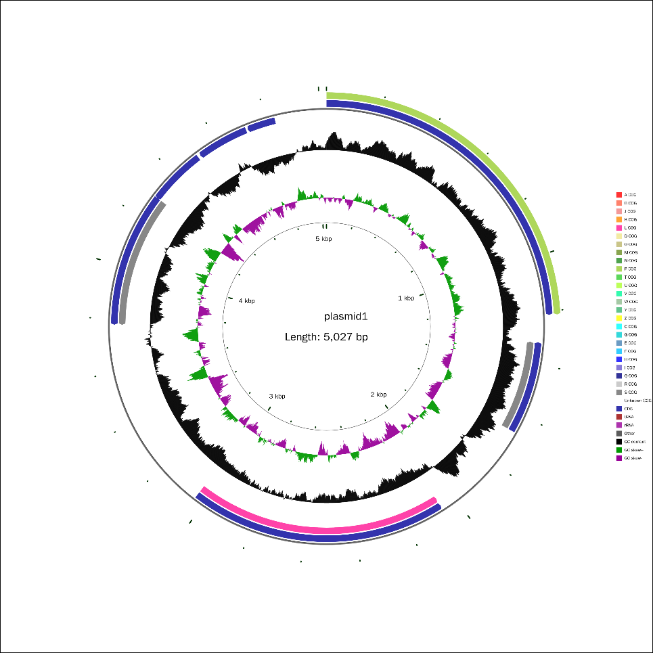


B

A


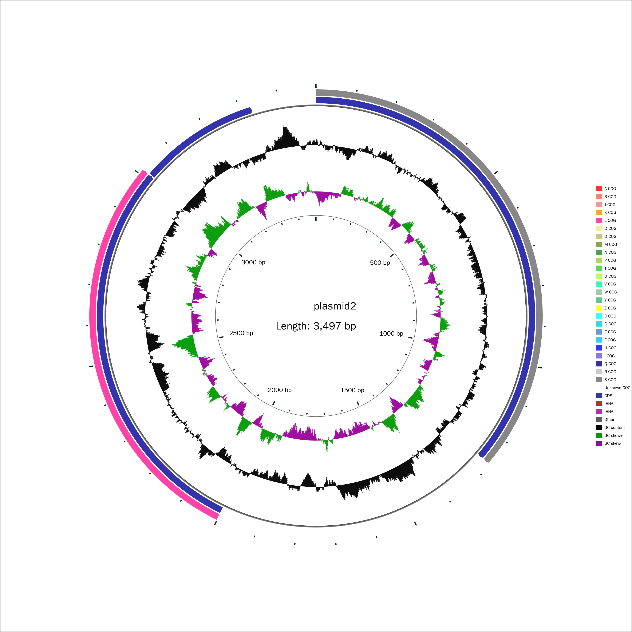


C

Note: (A) Gene map of the complete sequence for plasmid carried by multidrug resistance APP-R. This circular genome map was generated by CGView Server. From inside to outside, the circulars indicated reverse ORFs, GC content (%), GC skew, and forward ORFs. (B) Gene map of the complete sequence for resistance plasmid pGD2107-1 carried by florfenicol-producing APP-R. The florfenicol resistance genes (*floR*) were located in this plasmid. (C) Gene map of the complete sequence for other plasmid pGD2107-2 carried no resistance gene.

**Table S1. The resistance genes in *A.pleuropneumoniae* GD2107.**

| location | resistance genes |
| --- | --- |
| chromosome | *aph(3'')-Ⅰb、macA、macB 、sul3 、tet34、tet35、tetO 、parC、parE、gyrA、gyrB、 rpoB、rpoC、 dfrA3、hns、 tuf2、golS、folP、crp、cpxR* |
| pGD2107-1 | *floR* |
| pGD2107-2 | / |

**Table S2. The information on plasmids.**

| plasmid | Area | Host bacteria | coverage | similarity | length（bp） | ARG | Accession no. |
| --- | --- | --- | --- | --- | --- | --- | --- |
| pGD2107-1 | Guangdong | *A. pleuropneumoniae* |  |  | 5027 | *floR* | [CP097378.1](https://www.ncbi.nlm.nih.gov/nucleotide/CP097378.1?report=genbank&log$=nucltop&blast_rank=1&RID=1UPSVUDN016) |
| pMAF5 | Shanghai | *P.multocida* | 98 | 94.95 | 5279 | *floR* | [CP100664.1](https://www.ncbi.nlm.nih.gov/nucleotide/CP100664.1?report=genbank&log$=nucltop&blast_rank=37&RID=1UPSVUDN016) |
| pXL001 | Guangdong | *P.multocida* | 100 | 97.69 | 5047 | *floR* | CP077724.1 |
| pAPPJY | Jiangsu | *A. pleuropneumoniae* | 90 | 99.93 | 5119 | *floR* | [OP122556.1](https://www.ncbi.nlm.nih.gov/nucleotide/OP122556.1?report=genbank&log$=nucltop&blast_rank=46&RID=1UPSVUDN016) |
| pSC1810 | Sichuan | *A. pleuropneumoniae* | 100 | 97.5 | 10096 | *floR* | [CP071699.1](https://www.ncbi.nlm.nih.gov/nucleotide/CP071699.1?report=genbank&log$=nucltop&blast_rank=2&RID=1UPSVUDN016) |
| pIV86 | Fujian | *A.indolicus* | 98 | 95.82 | 5257 | *floR* | OQ325044.1 |
